# CHANGE IN ALZHEIMER’S DISEASE BLOOD-BASED BIOMARKERS AND ASSOCIATIONS WITH BRAIN AMYLOID DEPOSITION

**DOI:** 10.1093/geroni/igae098.0004

**Published:** 2024-12-31

**Authors:** James Pike, Yifei Lu, Jinyu Chen, Keenan Walker, Kevin Sullivan, Michael Griswold, Thomas Mosley, Priya Palta

**Affiliations:** New York University, New York, New York, United States; Merck, Landsdale, Pennsylvania, United States; University of North Carolina, Chapel Hill, North Carolina, United States; National Institute on Aging, Baltimore, Maryland, United States; University of Mississippi Medical Center, Jackson, Mississippi, United States; University of Mississippi Medical Center - The MIND Center, Jackson, Mississippi, United States; University of Mississippi Medical Center - The MIND Center, Jackson, Mississippi, United States; University of North Carolina, Chapel Hill, North Carolina, United States

## Abstract

Blood-based biomarkers show promise as a noninvasive, inexpensive method for measuring Alzheimer’s disease pathology throughout the lifecourse. However, the prospective associations and discriminatory accuracy of these biomarkers at different stages of the lifecourse and among diverse, community-dwelling populations requires further investigation. Between 2014 and 2015, 329 dementia-free participants from the Atherosclerosis Risk in Communities Study underwent brain MRI and PET scans. Amyloid positivity was defined as a standardized uptake value ratio greater than 1.2. Stored plasma samples collected in midlife (1993-95, mean age 58.5 years) and late-life (2011-13, mean age 76.2 years) from a subsample of 259 participants were assayed in 2022. The assay quantified amyloid-β (Aβ)42/40, phosphorylated tau (p-Tau181), neurofilament light (NfL), and glial fibrillary acidic protein (GFAP). Logistic regression models estimated the association between plasma biomarkers from midlife and late-life and amyloid positivity in late-life. Receiver operating characteristic curves documented the discriminatory accuracy of the biomarkers. Assays from midlife were not associated with amyloid positivity in late-life. However, late-life measurements of Aβ42/40, p-Tau181, and GFAP and change per decade from midlife to late-life in p-Tau181 and GFAP were associated with greater odds of amyloid positivity. The greatest discriminatory accuracy was achieved by using all assays measured in midlife and late-life (AUC = 0.754), although the accuracy when using only late-life measures was comparable (AUC = 0.737). Additional longitudinal research is needed to determine whether changes in plasma biomarkers measured before an individual is amyloid positive can identify older adults at risk of developing Alzheimer’s disease.

